# How to Amputate a Leg

**Published:** 1882-02-20

**Authors:** Theodore A. McGraw

**Affiliations:** Michigan; Professor of Surgery in the Detroit Medical College


### HOW TO AMPUTATE A LEG.

BY THEODORE A. MCGRAW, M. D.,

Professor of Surgery in the Detroit Medical College. Clinical Lecture delivered at St. Mary's Hospital on October 15th, 1881.

GENTLEMEN—Two years ago this young man, previously healthy, acquired constitutional syphilis. I do not know how he had been treated nor why the disease should have assumed so intractable a form. When he entered the hospital a month ago, he was suffering as he now is from caries of the left ankle and both right and left sternoclavicular articulations, from white swelling of the right elbow and from an ulcer in the back. Ninety grains of iodide of potassium have been given him every day for three weeks without avail in arresting the disease, and the ankle has become so intolerably painful and is so thoroughly disorganized that we have decided upon amputation as a means of saving his life. And simple as an amputation may seem to you, there are many things to consider. First of all, as we have the privilege in this case of electing our place of amputation, we have to ask whether the patient can afford an artificial leg, or whether he will have to content himself with a wooden peg. In the latter case we would serve his interests best by making a very short stump which would not project inconveniently far behind him nor be in his way. In the former case, we would make the stump longer so as to give a better support for the artificial leg, and yet not so long as to occupy all the space above the ankle. The mechanism which moves the ankle and other joints of the foot in an artificial leg is usually placed in a hollow part of the apparatus below the stump. It would be a mistake, therefore, to amputate so low down as to occupy all such available space and to force the artificer to use other and less suitable expedients for accomplishing the purpose In this case, I will choose the middle third of the leg, and make a stump to which either a peg or a leg may be adapted as circumstances may require. In the next place I will endeavor with this anemic patient to save every drop of blood, and for that purpose will use an Esmarch bandage, beginning to wind it, however, above the seat of disease in order that no pus nor putrid material may be forced into the circulation. By means of this bandage the circulation can be perfectly controlled and all the main arteries and veins of the leg be tied without any hemorrhage whatever. This does not, however, always make a perfectly bloodless operation, for you will often find that the removal of the constricting cord will be followed by quite a stream of blood, part of which regurgitates through the open mouths of the united veins and part of which flows from anteries which have escaped notice. This is especially the case with robust plethoric patients and where long standing irritations have produced enlargement of the collateral vessels. Now that the patient is ready, we will apply the bandage and prepare for operating. I will make two skin flaps, each of which consist of the skin of half the circumference of the leg and about three inches long. These being retracted without pulling the patient down upon the table at all, I cut the muscles through with this long narrow knife, passing the blade between the bones and cutting the flesh entirely away.

I wish you to notice particularly how easy it is to amputate a leg quickly and neatly without disturbing the position of the patient on the table. My assistant throws the limb to the side of the table and I apply the saw to the fibula first, and then to the tibia. You see that I dispense entirely with the three tailed retractor. Commonly used it is altogether unnecessary, and takes up unnecessary time. Now that the leg is off, I take up first the anterior and then the posterior arteries, and after that their respective veins. Upon loosening the constricting cord, however, you see a free hæmorrhage which requires further ligation of veins and arteries. I will now wash the stump with hot water and bring the flaps together with sutures, having first put a rubber drainage tube through the wound. In dressing the stump we will press the flaps firmly on the cut surfaces and bandage them snugly. Carbolized oakum being first applied as an elastic compress.

Now that the patient is removed, I wish to call your attention again to two points in which my procedure differs from that of the books. 1st. I did not draw the patient off the table. This habit dates from the days before anesthesia, when the skill of the surgeon was reckoned inversely as the time occupied in operating. Nowadays with unconscious patients, it is far easier and altogether neater and less confusing to draw your patient near the side of the table, to have the leg elevated by an assistant, and to proceed quietly with the operation. Why should one hurry when a moment's delay causes neither shock, pain nor loss of blood?

2nd. I dispensed with the altogether unnecessary retractor. I might say in addition a word of warning, and that is, to avoid a method of operating which also dates from an ancient time. Never amputate a leg by making a posterior flap from the calf. Such flaps can be made with the utmost celerity but their weight makes them afterwards unmanageable. They drag away from their anterior attachments and rarely heal by first intention. I have seen multitudes of such stumps, and I have never yet seen one that seemed to me satisfactory when [illegible] ealed.—[Detroit Clinic.

